# Network pharmacology and in vivo experimental analysis for validating the antifibrotic potential of *Punica granatum* leaves against skin scleroderma: the role of oxidative stress, inflammation and TGF-β1/Snail 1/p-Smad 3 signaling pathway

**DOI:** 10.1007/s10787-026-02245-y

**Published:** 2026-04-27

**Authors:** Sara M. Baraka, Zeinab A. El-Gendy, Eman A. W. El-Abd, Ahmed F. El-Sayed, Enayat A. Omara, Marwa M. Elbatanony

**Affiliations:** 1 Chemistry of Natural Compounds Department, National Research Centre, Giza, 12622 Egypt; 2 Department of Pharmacology, Medical Research and Clinical Studies Institute, National Research Centre, Dokki, Giza, Egypt; 3 Pharmacognosy Department, National Research Centre (NRC), El Behouth St., P.O. 12622, Cairo, Egypt; 4 Microbial Genetics Department, Biotechnology Research Institute, National Research Centre, Dokki, Giza, 12622 Egypt; 5 Egypt Center for Research and Regenerative Medicine (ECRRM), Cairo, Egypt; 6 Pathology Department, National Research Centre, Dokki, Cairo Egypt

**Keywords:** LC/MS, *Punica granatum*, Network pharmacology, Skin fibrosis, TGF-β pathway

## Abstract

Given the significant efficacy of the *Punica granatum* in enhancing skin health, this research employed network pharmacology and in vivo studies to explore the *P. granatum* ethanolic leaf extract’s (PGEL) mechanism in alleviating skin sclerosis. A model of skin fibrosis brought on by bleomycin (100 µl/rat, sc.) was adopted for experimental validation, where PGEL was orally administered at 200 and 400 mg/kg to rats for 3 weeks. Seventy-three compounds of PGEL were identified by LC/MS/MS belonging to different chemical classes; an organic acid, 12 phenolic acids, 4 polysaccharides, 3 amino acids, 19 gallic acid derivatives,7 ellagic acid derivatives, 14 flavonoides, three anthocyanins, 3 fatty acids and 7 miscellaneous groups. Five compounds (Catechin-3-O-gallate, 3,3′-di-O-methyl-4-O-(xylopyranosyl) ellagic acid, ellagic acid glucoside, valoneic acid dilactone, and vitexin 2-O-gallate) were isolated from the PGEL. Network pharmacological studies clarified that TNF-α, TGF-β1, Snail1, p-Smad3, MMP-9, IL-17 A, and COL1A1 are the core targets for skin fibrosis. PGEL lessened MPO and increased SOD activity in rat’s skin, underscoring its antioxidant activity. Furthermore, the TNF-α, IL-17 A and MMP-9 were decreased in PGEL groups. Mechanistic studies revealed that PGEL exerts its anti-fibrotic action by downregulating the TGF-β1, Snail 1, COL1A1, and p-Smad 3 in the skin tissues. Additionally, histopathological analyses informed the decline in dermal alterations and thickness in rats treated with PGEL. Collectively, our results give clear evidence that PGEL work through multi-pathway modulation by targeting the core proteins of inflammation mediated the TGF-β/Snail1/Smad 3 signaling, thereby exerting a therapeutic action on skin fibrosis.

**Graphical abstract:**

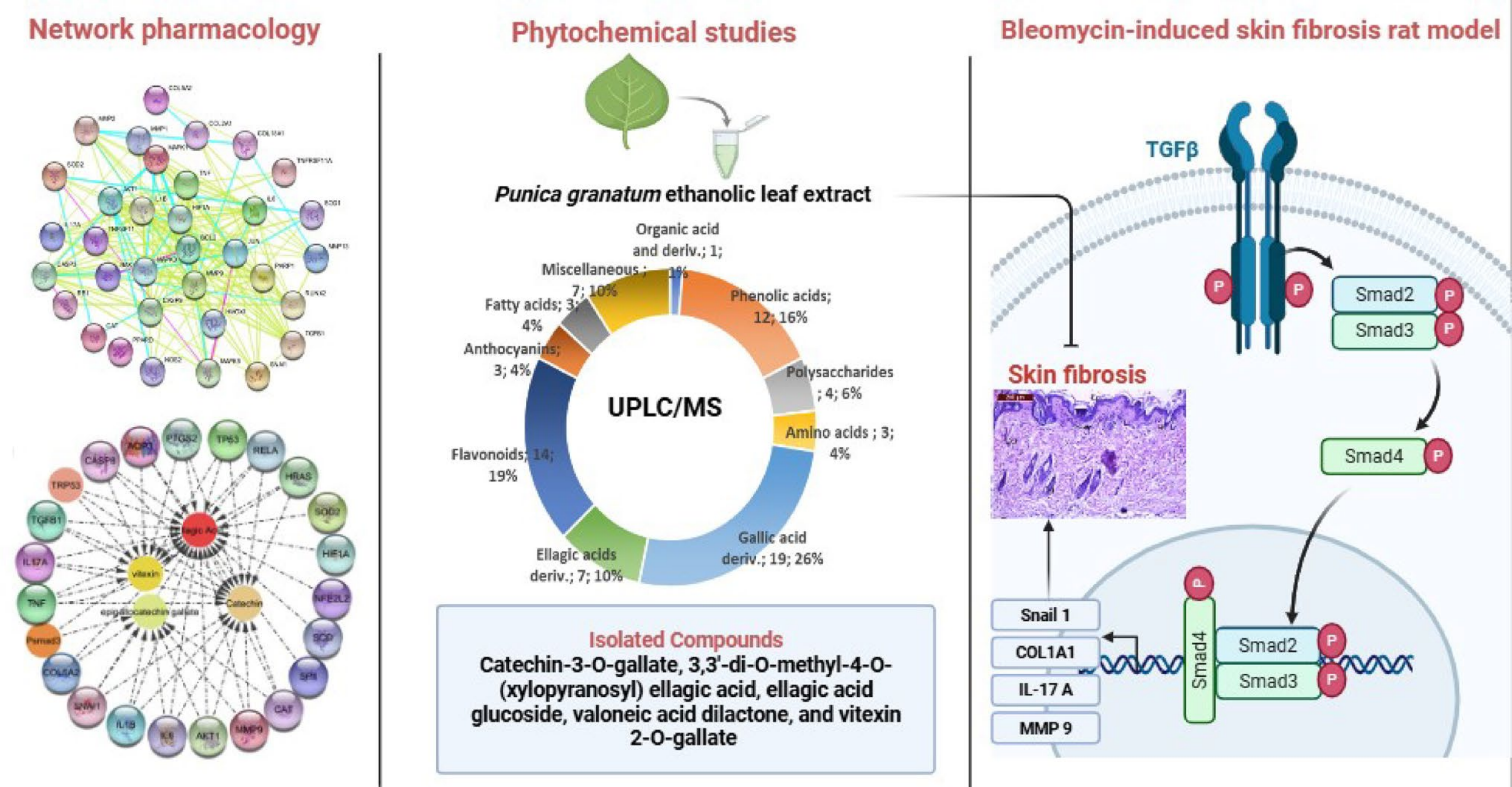

## Introduction

Tissue fibrosis brought on by an overabundance of collagen and other extracellular matrix (ECM) constituents in the skin and visceral organs is a hallmark of systemic sclerosis (SSc), an autoimmune illness (Distler et al. 2019). The skin can develop fibrosis in response to local stimuli, but also, though less frequently, as a result of systemic inflammatory conditions. SSc, also known as scleroderma, exemplifies a condition where inflammation linked to autoimmunity and extensive vascular dysfunction becomes dysregulated, leading to pathological fibrosis in the skin and internal organs (Asano 2018). Localized skin fibrosis occurs in keloids, hypertrophic scars, Morphea, and numerous other conditions with vascular, metabolic, or genetic origins (Jinnin 2010). The overproduction and tissue deposition of ECM in SSc is mostly caused by activated fibroblasts and myofibroblasts. Numerous pro-fibrotic molecules, such as transforming growth factor-β (TGF-β), interleukin-17 A (IL-17 A), and matrix metalloproteinase 9 (MMP-9), trigger a complex cascade of events that culminate in fibroblast stimulation and the phenotypic shift to myofibroblasts (Asano 2018).

Among these, TGF-β is probably the most important molecule for the local fibroblasts’ functional activation and the tissue fibrosis that results in SSc (Lafyatis 2014). Activated TGF-β binds to its cell surface receptor, causing both canonical and noncanonical Smad-independent pathways to undergo intracellular signal transduction. Smad2 and 3 become phosphorylated when TGF-β receptor type I is activated, which enables it to mix with Smad4 and go into the nucleus where it attaches to Smad-binding element sequences of TGF-β sensitive genes (Frangogiannis 2020). ECM deposition and tissue fibrosis can be caused by excessive or dysregulated TGF-β/Smad signaling (Frangogiannis 2020). These abnormal alterations include the loss of redox control, which results in oxidative stress and hypoxia, as well as the production of pro-inflammatory cytokines and aberrant angiogenesis/vasculogenesis regulators (Jiang et al. 2020). In fact, bleomycin-induced SSc animal’s dermal fibroblasts have been shown to exhibit persistent signal activation (Takagawa et al. 2003), and in experimental SSc models, deactivating the signaling cascade reduces skin fibrosis (Utsunomiya et al. 2022; Wei et al. 2014).

The historical record contains extensive documentation of the use of medicinal products made from various herbal species to treat dermatological conditions (Papp et al. 2022). Dermatological conditions are the fourth most common contributor to human diseases. The Global Burden of Disease Study has documented that around one-third of the global population suffers from at least one skin disease, and the estimated burden of these conditions is substantial in both high- and low-income nations (Hay et al. 2014). Due to their demonstrated beneficial effects on the skin, as shown in previous endeavors, natural products and their active compounds have attracted considerable attention in dermatology and cosmetology (Kumar et al. 2023). Pomegranate (*Punica granatum* L.), family Punicaceae, is native to the Mediterranean areas. All pomegranate plant parts, including peels, fruit, seed, flower, and leaves, provide a diversity of therapeutic activities (Ranjha et al. 2023). It is considered as a rich source of polyphenolics, with a wide range of diverse classes (phenolic acids, hydrolyzable tannins, and flavonoids) (Mena et al. 2012). A recent study investigated hydrolysable tannins (ellagitannins) of *P. granatum* peels where punicalin, punicalagin in addition to gallic, caffeic and protocatechuic acids and other flavonoids were identified and isolated (El Sawi et al. 2024). *P. granatum* leaves are rich source of flavonoids, anthocyanins, tannins, phenolics. They were proved to exert potential effect on cholesterol management and body weight loss (Wang et al. 2018), diabetic nephropathy (Mestry et al. 2017), as well as anti-inflammatory, anti-dandruff, aiding hair growth (Bhinge et al. 2021), and anti-cancer properties (Fakudze et al. 2024). In addition, the non-polar extract of *P. granatum* leaves proved its antimicrobial activity against different pathogens as well as it showed no toxic effect on skin fibroblast normal cells (BJ1) (Elbatanony et al. 2019). Various *P. granatum* extracts possesses beneficial dermatological effects on skin aging, erythema, pigmentation, striae distensae, and psoriasis (Dimitrijevic et al. 2024). In addition, Kasai et al. (Kasai et al. 2006) validated the ameliorative actions of ellagic acid-rich pomegranate extract against ultraviolet irradiation-induced pigmentation in the human skin. Hence, *P. granatum* suggests promising avenues for its prospective application in managing multiple skin lesions.

LC-ESI-MS, a mainstream tool with high accuracy, provides valuable insights to identify the phyto-constituents in plant extracts. Thus, to provide a detailed profile of the compounds in *P. granatum* ethanolic leaf extract (PGEL), this technology has been employed. Simultaneously, network pharmacology studies have been conducted to speculate on the target keys-mediated skin diseases. Therefore, this research has been established to explore the potential targets and signaling pathways of PGEL to alleviate skin sclerosis. Additionally, the main targets and pathways that mediated fibroblasts activation were verified through in *vivo* trials by adopting the model of bleomycin-induced skin fibrosis in rats.

## Materials and methods

### Phytochemical studies

#### Plant material

The fresh *P. granatum* leaves were collected from National Agriculture Centre (Egypt), cleaned off from the impurities and shade dried. It was kindly identified by Dr. Gamal Farag, National Agriculture Centre (NRC), Giza, Egypt. A voucher sample of leaves were deposited at the NRC herbarium and given a number of M216.

#### Plant extraction

The air-dried powdered (300 g) leaves were extracted on cold by ethanol (80%) three times (7 L of ethanol were consumed in total). The yielded extracts were combined, evaporated and condensed to dryness under vacuum at 40 °C, weighted and kept in refrigerator for biological and chemical studies.

#### Chemicals

All chemicals and solvents were of high analytical grade, products of Merck, Germany and Sigma (USA).

#### Instrumentals

Nuclear Magnetic Resonance spectrometers JEOL EX-270 MHz, 300 MHz and 125 MHz for determination of ^1^H-NMR and ^13^C-NMR, rep., Mass spectrometer; Finnigan Model 3200 at 70 eV.

#### LC/MS-MS

The analysis of the ethanol fraction was performed using liquid chromatography–electrospray ionization–tandem mass spectrometry (LC-ESI-MS/MS) with an ExionLC (High flow LC) Hardware for chromatographic separation and SCIEX Triple for separation and SCIEX Triple Quad 5500 + MS/MS system equipped with an electrospray ionization (ESI) for detection (El Sayed et al. 2020).

#### Flavonoid and phenolics isolation

The dried ethanol (80%) extract (PGEL, 35 g) was applied on TLC silica gel GF_254_ (Merck, 20 × 20 cm) plates using solvent systems of CHCl_3_: CH_3_OH (9:1 v/v: ). The bands have been observed under UV 254 and 365 nm. The existence of flavonoids and phenolics was established after spray with 1% ethanolic AlCl_3_ and FeCl_3_. All the isolated compounds have been purified on preparative TLC and identified by diverse spectral analyses (mass spectrometry, ^1^H -NMR and ^13^C-NMR). Each isolated compound was weighed in milligrams using a high-sensitivity analytical balance to estimate its corresponding amount in the dried extract.

### Network pharmacology

#### Screening of potential therapeutic targets for bioactive compounds

The SMILES codes or SDF files of compounds were obtained from the PubChem database. The SwissTargetPrediction database was used to identify the targets of these compounds, with “Homo sapiens” set as the screening criterion to pinpoint potential therapeutic targets. The DAVID database was employed to standardize and convert target names. To identify relevant targets, multiple databases—GeneCards, DrugBank, the Comparative Toxicogenomics Database (CTD), and DisGenest—were systematically searched using the SMILES codes or SDF files of the compounds. The objective was to compile a list of disease-related targets for further analysis. Duplicate entries were eliminated to create a comprehensive set of compound-associated targets.

#### Gene Ontology (GO) enrichment and KEGG pathway analysis

After identifying overlapping targets across multiple databases, GO functional enrichment and KEGG pathway analyses were conducted on these intersecting genes using the DAVID database (david.ncifcrf.gov). The analysis was limited to Homo sapiens with a significance threshold of *P* < 0.01. The top 10 entries for each category—cellular component, biological process, and molecular function—along with the top 20 pathways, were visualized and further evaluated using an online bioinformatics platform (http://www.bioinformatics.com.cn).

#### Protein–protein interaction (PPI) and drug-target network construction

To identify shared targets between bioactive compounds and skin disease-related targets, Venn diagrams were created using the Venny tool. https://bioinfogp.cnb.csic.es/tools/venny/. These common targets were then entered into the STRING database to build a protein–protein interaction (PPI) network, with analysis parameters set for “Homo sapiens” and a confidence threshold greater than 0.9. Unconnected nodes were excluded, while other parameters were kept constant, resulting in a PPI network relevant to compound-skin disease interactions. The network was visualized and analyzed using Cytoscape version 3.9.1 (cytoscape.org). To further investigate the mechanisms of potential therapeutic targets of bioactive compounds, Cytoscape 3.9.1 was used to construct and visualize a drug-target network.

### In vivo studies

#### Materials

Bleomycin utilized in this research was acquired as Bleocel 15 IU (15 mg) injection manufactured by CELON Labs (India), and dissolved in sterile saline at concentration of 2 mg/2 ml. All other utilized reagents were in high analytical grade. The PGEL was dissolved in a few amounts of tween 80 and completed with distilled water, giving the adopted concentration.

#### Animals and ethical approval

Male Wistar rats (160–180 g) were employed for the in vivo study. The rats were sourced from the animal house colony, National Research Centre (Giza, Egypt) and kept there under particular pathogen-free conditions at ambient temperature, and 12 h/12 h light/dark cycle with unlimited access to food and water. This research was performed in compliance with the ethical consideration of the ARRIVE guidelines and the instructions established by Ethical Conduct in the Care and Use of animals (NIH Publications No. 8023, revised 1978). This experiment has been approved by the Medical Ethics Committee (MREC) at National Research Centre (Giza, Egypt) under approval number 04410524. No mortality cases were recorded during the experiments. At the end of the experiment, the rats were expertly euthanized by cervical dislocation for skin tissue harvesting.

####  Bleomycin-induced dermal fibrosis rat model

Prior to the experimental studies, the animals were acclimatized for a week. All rats were depilated at the injection site by shaving a 2 cm^2^ area on the upper dorsal skin, centered over the thoracic region, using an electric clipper. The shaved area was gently cleaned to remove any residual hair without applying a razor or chemical depilatory agents to prevent potential skin irritation or barrier disruption. The following injection protocol has been employed: the first four subcutaneous injections were located at the corners of the 2 cm^2^-square shaved area and used in rotation (1→2→3→4), while the site of fifth injection was located in the middle of the square.

The rats were randomly allocated into four groups (*n* = 6/group): Normal group; rats received subcutaneous injection of saline (100 µl/rat) every 2 days for a total of 10 injections over 21 days at the upper dorsal area of back shaved skin. In BLEO group: rats were injected s.c. with bleomycin (100 µl / rat, 0.1 IU) every 2 days for a total of 10 injections over 21 days at the upper dorsal area of back shaved skin following the protocol of Roh et al. (2021). BLEO + PGEL 200 and BLEO + PGEL 400 groups: in these groups, rats orally administered with 200 and 400 mg/kg bw PGEL/day/21 days (Viswanatha et al. 2019) respectively with concurrent injection of bleomycin as in the second group.

Rats were sacrificed under anesthesia (intraperitoneal injection of sodium phenobarbital 150 mg/kg bw), after the last injection of bleomycin or saline. Skin tissues were individually harvested. For the histopathological assessments, skin parts were fixed in 10% neutral buffered formalin. Other portions of skin tissues were stored at − 80 °C for the molecular and biochemical investigations.

#### Biochemical analyses

##### Production of skin tissue homogenate

The skin specimens for each group was individually extracted by a 0.05 M phosphate-buffer saline (pH = 7) at 1:10 (w/v) by a Polytron homogenizer. Followed this, a centrifugation step was accomplished by a cooling centrifuge (Laborezentrifugen, 2k15, Sigma, Germany) for 10 min at 10,000 rpm. The isolated supernatant was stored at − 80 °C for further biochemical analyses. Following the method of Bradford (Bradford 1976), the content of total protein was estimated in the supernatant.

##### Evaluation of antioxidant and oxidant biomarkers

For estimation of antioxidant status of skin tissue, the activity of superoxide dismutase (SOD) was determined using a kit obtained from Santa Cruz Biotechnology, Inc., USA. On the other hand, myeloperoxidase (MPO, specific indicator of oxidative insult) level was quantified in the skin tissue homogenate by rat ELISA kit supplied by BioVision, USA, catalog No: E4581-100.

##### Evaluation of inflammatory and fibrotic biomarkers

Using rat ELISA kits, the levels of IL-17 A (Elabscience®, USA, Catalog No: E-EL-R0566), TNF-α (BioLegend, San Diego, California, Catalog No: 438204), MMP-9 (Fine Test®, Wuhan, Hubei, China, Catalog No: ER0139 ), TGF-β1 (Fine Test®, Wuhan, Hubei, China, Catalog No: ER1378), and p-Smad 3 (Antibodies, USA) were assessed in the skin tissue homogenate.

#### Molecular investigations (Quantification of COLA1 and Snail 1 mRNA expression)

The skin samples were processed for RNA extraction utilizing a kit (NucleoSpin® REF. 740901.250) supplied by Macherey- Nagel GmbH & Co. Germany. A kit was purchased from by Bioline, a median life science company, UK (SensiFAST™ SYBR® Hi-ROX One-Step Kit, catalog no.PI-50217 V) to achieve the step of quantitative real time –polymerase chain reaction (qRT-PCR). Table 1 presented the sequence of primer of each target genes (COLA1 & Snail1) and reference housekeeping gene (GAPDH; glyceraldehyde-3-phosphate dehydrogenase).The relative quantitation (RQ) of each target gene is quantified according to the calculation of delta-delta Ct (ΔΔCt). Then, by taking 2^−∆∆Ct^, the RQ of each gene was computed.

**Table 1:** Primers sequence of the studied genes

Gene symbol	Primer sequence from 5′- 3′
*COLA1*	F: GTACATCAGCCCAAACCCCAAG R: CGGAACCTTCGCTTCCATACTC NM_053304.1
*Snail1*	F: TGCACGACCTGCGAAAG R: TGTGGAGCAAGGACATTCG XM_032902473.1
*GAPDH*	F: CACCCTGTTGCTGTAGCCATATTC R: GACATCAAGAAGGTGGTGAAGCAG NM_001394060.2

#### Histopathological and morphometric examination

The skin specimens were obtained from rat fixed in 10% buffered formalin, dehydrated in ascending grades of ethanol concentration, cleaned in xylol and embedded in paraffin. Skin sections of 5 μm thickness were cut and then mounted on glass slides. Paraffin sections were stained with Hematoxylin and Eosin to show the histological structures. The dimension of skin thickness was estimated indirectly by processing image J software, developed by the National Institute of Health (NIH, USA).

In addition, Masson’s trichrome stain was conducted to determine the content of collagen fibres.

### Statistical analysis

All data are presented as means ± standard error. To ensure a Gaussian distribution, the Shapiro-Wilk’s normality test was utilized, followed by a Tukey’s post-test for pairwise comparisons. The statistical significance level was established at *p* < 0.05 (95% CI). GraphPad Prism version 6.0 for Windows, GraphPad Software, San Diego, California, USA, was used to obtain statistical data and figures.

## Results

### Phytochemical analyses

#### LC/ESI MS results

Seventy-three compounds were tentatively identified by LC/MS/MS belonging to different chemical classes (Table 2); an organic acid, 12 phenolic acids, 4 polysaccharides, 3 amino acids, 19 gallic acid derivatives,7 ellagic acid derivatives, 14 flavonoides, three anthocyanins, 3 fatty acids and 7 miscellaneous groups. According to available literature, thirty-seven compounds were previously detected from *P. granatum* different parts. It is to be noted that 26 compounds, which represent the majority of the identified compounds, belong to hydrolysable tannins that comes in compatibility with literature (Fischer et al. 2011; Mena et al. 2012; Singh et al. 2016; Wu et al. 2022). In this study, 19 gallotannins (peaks 21–39) were identified as monogalloylglucose (7 compounds), digalloyl-glucose (4 compounds), a trigalloyl-glucose, polygalloyl‐glucose (4 compounds) and 3 galloyl-hexahydroxydiphenoyl (HHDP) gallic acid derivatives, in addition to 7 detectable ellagitannins (peaks 40–46). They demonstrate distinctive fragmentation pattern including loss of one or more galloyl moieties according to each structure. e.g. compound no.30, pedunculaginⅡ, showed at Rt 8.10, [M-H]^−^ 784.78861[C_34_H_25_O_22_]^−^, yielded fragments at *m/z*: 633, 623 for the loss of galloyl and glucose, respectively, followed by 301 which was attributed to the loss of the 2 remaining galloyl moieties and CO group (Hooi Poay et al. 2011; Mena et al. 2012; Singh et al. 2016).

Among identified flavonoids, 2 compounds made characteristic conjugation with gallic acid: Vitexin 2’’-O-gallate (peak no. 54) at *m/z* gave 584.9671 and other fragments were noticed at *m/z*: 432[M-galloyl], 342[M-galloyl-90], 312[M-galloyl-120] and 270 while, myricetin-3-O-gallate (peak no. 59), at *m/z* 469.0412, formed fragment ions at *m/z* 317, 169 corresponding to the loss of galloyl group and aglycone, respectively (Singh et al. 2016). Other flavonoid glycosides (Peaks: 47, 50–53 and 56) exhibited the classic pattern of fragmentation by loss of hexoside (162 Da) pentoside (146 Da), water (18 Da) or any other attached groups (Farid et al. 2022).

Among identified flavonoids, 2 compounds made characteristic conjugation with gallic acid: Vitexin 2’’-O-gallate (peak no. 54) at *m/z* gave 584.9671 and other fragments were noticed at *m/z*: 432[M-galloyl], 342[M-galloyl-90], 312[M-galloyl-120] and 270 while, myricetin-3-O-gallate (peak no. 59), at *m/z* 469.0412, formed fragment ions at *m/z* 317, 169 corresponding to the loss of galloyl group and aglycone, respectively (Singh et al. 2016). Other flavonoid glycosides (Peaks: 47, 50–53 and 56) exhibited the classic pattern of fragmentation by loss of hexoside (162 Da) pentoside (146 Da), water (18 Da) or any other attached groups (Farid et al. 2022).

**Table 2:** LC-ESI-MS/MS analysis of *Punica granatum* leaves ethanolic extract

No.	Rt.	(M-H) m/z	Fragments	M.F. (M-H)	Identified compounds	References
*Organic acid and its derivatives*						
1	1.71	132.9756	115, 101	C_4_H_5_O_5_^−^	malic acid	Ammar et al. (2023)
*Phenolic acid and its derivatives*						
2	1.72	191.0491	173[M-H- H_2_O], 145[M-H-CO], 111	C_7_H_11_O_6_^−^	Quinic acid	Wu et al. (2022)
3	1.79	168.9924	151[M-H- H_2_O], 125[ M-H-CO_2_],107	C_7_H_5_O_5_^−^	Gallic acid	Wu et al. (2022)
4	1.97	300.9615	283[M-H- H_2_O], 257 [M-H- CO_2_], 254, 229	C_14_H_5_O_8_^−^	Ellagic acid	Singh et al. (2016, Wu et al. (2022)
5	1.82	179.0552	161[M-H-H_2_O],135[M-H-H_2_O-CO]	C_9_H_7_O_4_^−^	Caffeic acid	Wu et al. (2022)
6	2.54	163.0173	145,119,101	C_9_H_7_O_3_^−^	*p*- Coumaric	Wu et al. (2022)
7	3.47	340.9788	179[M- H- hexose], 161[M- H- hexose-H_2_O], 161, 135	C_15_H_18_O_9_	Caffeic acid hexoside (Hydroxycinnamic acid derivative)	Fischer et al. (2011)
8	6.34	325.0067	163 [M-H- glucose]	C_15_H_17_O_8_-	*p*-Coumaric acid glucoside	El-Aguel et al. (2022; Wu et al. (2022)
9	8.26	463.0006	301[M-H- hexose],271	C_20_H_15_O_13_^−^	Ellagic acid hexoside	El-Aguel et al. (2022)
10	8.51	353.0661	309[M-H- CO_2_]161,159	C_16_H_17_O_9_^−^	Caffeoyl quinic acid	El-Aguel et al. (2022)
11	8.92	432.9614	301[ M-H- pentose],300	C_19_H_13_O_12_^−^	Ellagic acid-pentoside	Singh et al. (2016)
12	12.5	207	192,177,133	C_11_H_11_O_4_^−^	Sinapaldahyde	El Sayed et al. (2020)
13	14.53	153.0880	135[M-H- H_2_O], 121, 115, 109	C_7_H_5_O_4_-	Protocatechuic acid	Fischer et al. (2011)
*Polysaccharides*						
14	1.81	195.0327	177.01, 159, 129, 111, 75	C_6_H_12_O_7_^−^	Gluconic acid	Ammar et al. (2023)
15	1.82	179.0305	161 [M-H-H2O], 143	C_6_H_11_O_6_^−^	Fructose	Wu et al. (2022)
16	2.81	181.02742	163[M-H-H_2_O], 131[M-H-H_2_O-CH_2_], 119, 101	C_6_H_13_O_6_^−^	Mannitol	Wu et al. (2022)
17	3.37	134.9272	117[M-H-H_2_O], 107 [M-H- CO]	C_4_H_7_O_5_^−^	Threonic acid	Wu et al. (2022)
*Amino acids*						
18	1.90	128.0220	128	C_5_H_6_NO_3_^−^	4-Oxoproline	Wu et al. (2022)
19	3.08	203.0828	159 [M-H-CO_2_],142[ M-H-CO_2_-NH3],116	C_11_H_11_N_2_O_2_^−^	Tryptophan	Wu et al. (2022)
20	3.48	146.9228	129[M-H-H_2_O] 103 [M-H- CO_2_]	C_5_H_8_NO_4_-	Glutamic acid	Wu et al. (2022)
*Hydrolyzable tannins*						
A) Gallic acid derivatives						
21	2.10	331.0448	271, 169 [M-H- glucose]	C_13_H_15_O_10_^−^	Glucogallin (monogalloyl-glucose)	Wu et al. (2022)
22	2.87	483.0782	331,313,169,125,315,297,	C_20_H_19_O_14_^−^	Digalloyl-glucoside	Wu et al. (2022)
23	5.47	634.91184	466 [M-H- galloyl], 169 [gallic acid]	C_27_H_23_O_18_^−^	Corilagin	Wu et al. (2022)
24	6.03	630.83	613[M-H- H_2_O] ,463 ,445,301,169	C_27_H_19_O_18_^−^	HHDP glucoside derivative [(-)-vescalin; castalin	Wu et al. (2022)
25	6.91	468.9521	451(M-H-H_2_O), 441(M-H-CO), 317 (M-H-galloyl)	C_21_H_9_O_13_-	Valoneic acid dilactone	Wu et al. (2022)
26	7.13	932.7866	781[ M-H- galloyl], 273, 301	C_41_H_25_O_26_-	Galloylpunicalin	Wu et al. (2022)
27	7.38	781.7511	763[M-H- H_2_O], 753 [M-H-CO], 751[M-H-OCH_2_], 721[M-H-2OCH_2_], 629 [ M-H- galloyl 152]	C_34_H_21_O_22_^−^	Punicalin	Fischer et al. (2011, Wu et al. (2022)
28	7.38	782.8511	765[ M-H-H_2_O], 631[M-H- galloyl], 451, 301	C_34_H_23_O_22_^−^	Granatin A	Singh et al. (2016, Wu et al. (2022)
29	8.03	786.82465	634 [M-H- galloyl], 616[M-H- galloyl- H_2_O], 449	C_34_H_27_O_22_-	1,2,4,6-tetra-O-galloyl-glucose	Wu et al. (2022)
30	8.10	784.78861	633 [M-H- galloyl], 623 [M- H- glucose], 301[ M-H- 2 galloyl-CO]	C_34_H_25_O_22_^−^	PedunculaginⅡ	Wu et al. (2022)
31	8.16	452.9579	423[M-H- CH2O],313, 291[ M-H- lucose], 169[ gallic acid]	C_20_H_21_O_12_^−^	3-Methoxy-4-hydroxyphenol-(O-galloyl)-glucoside	Singh et al. (2016)
32	9.56	196.9974	169[ gallic acid moiety], 125[ gallic acid –CO_2_], 78	C_9_H_9_O_5_-	Ethyl gallate	Singh et al. (2016)
33	10.01	331	271[M- H- C_2_ H_4_O_2_], 211[M- H- 2 C_2_H_4_O_2_], 169 [M-H- hexose]	C_13_H_15_O_10_^−^	Galloyl hexoside (Glucogallin)	Wu et al. (2022)
34	10.91	477.0198	325[M-H- galloyl],313 ,169 [gallic acid], 125	C_21_H_17_O_13_^−^	Digalloyl-shikimic acid	Singh et al. (2016)
35	12.44	649.1716	605 [M-H- CO_2_], 479 [M-H- galloyl], 481	C_27_H_23_O_18_^−^	Trisgalloyl glucose	Mena et al. (2012)
36	16.42	633.1662	481[M-H- galloyl], 463[ 481 Da- H_2_O], 301[ 463 Da- hexose], 249	C_27_H_37_O_17_^−^	Galloyl-HHDP-hexose	Fischer et al. (2011, Singh et al. (2016)
37	17.11	457.1596	305[M-H- galloyl], 169, 125	C_22_H_17_O_11_^−^	Gallocatechin-3-O-gallate	Singh et al. (2016)
38	22.96	950.7617	907[ M-H-CO_2_], 799[ M-H- galloyl], 781[ M-H- gallic acid], 629[781 Da- galloyl], 477[ 629 Da- galloyl], 459[477 Da-H_2_O],301[ ellagic acid]	C_41_H_27_O_27−_	Trisgalloyl HHDP glucose	Singh et al. (2016)
39	23.82	441.0842	289[M-H- galloyl], 245[M-H- galloyl-CO_2_], 169	C^22^H^17^O^10^-	(+)-Catechin-3-O-gallate	Singh et al. (2016)
B) Ellagic acid derivatives						
40	8.09	950.7251	932[M-H- H_2_O], 914[932Da- H_2_O], 613[ M- H-2H_2_O –ellagic acid], 301	C_41_H_27_O_27_^−^	Granatin B	Fischer et al. (2011)
41	8.09	936.7456	768[M-H- C_7_H_5_O_5_], 301[ellagic acid]	C_41_H_29_O_26_-	Punicafolin	Wu et al. (2022)
42	8.84432	544.8994	485[M-H-H_2_O- COCH_2_], 470 [M-H-H_2_O- COCH_2−_ CH_3_], 383 [ M-H- hexose]315, 301	C_25_H_21_O_14_^−^	Methylellagic acid-di-acetyl-hexoside	Singh et al. (2016)
43	9.95	461.0449	446[M-H-CH3], 329 [M-H- pentose], 314[M-H- pentose-CH3], 298	C_21_H_17_O_12_-	3,3’-Di-O-methyl-4-O-(xylopyranosyl) ellagic acid	Singh et al. (2016)
44	9.99	598.9072	447[ M-H- galloyl], 301, 169[gallic acid]	C_27_H_19_O_16_-	Galloyl-rhamnopyranosyl) ellagic acid	Singh et al. (2016)
45	10.89	328.9393	314[ M-H- CH_3_], 299 [ M-H- 2CH_3_]	C_16_H_9_O_8_-	di-O-Methyl ellagic acid	Singh et al. (2016)
46	14.09	343.0567	328 [M- H- CH_3_], 313[M- H- 2CH_3_], 298[M- H-3 CH_3_] 285, 269	C_17_H_11_O_8_-	Tri-O-methylellagic acid	Singh et al. (2016)
*Flavonoids and derivatives*						
47	7.97	609	447[M-H- hexose], 301 [M-H- hexose- pentose], 285	C_27_H_29_O_16_^−^	Quercetin-3-O-rutinoside (Rutin)	Wu et al. (2022)
48	8.27	300.9826	283 (M-H-H2O),257,161,151	C_15_H_9_O_7_-	Quercetin	Yaritz et al. (2022)
49	8.35	305.0712	287 [M-H- H_2_O], 269[M-H- 2H_2_O], 261[M-H- CO_2_]	C_15_H_13_O_7_-	Gallocatechin	Mena et al. (2012), Wu et al. (2022)
50	8.48	463	301,283,271	C_21_H_18_O_12_^−^	quercetin-3- O-glucoside	Wu et al. (2022)
51	9.22	447.0988	285[ M-H- hexose], 255 [M- M-H- hexose CH_2_O],151	C_21_H_19_O_11_^−^	Kaempferol-O-hexoside	Wu et al. (2022)
52	9.81	430.9656	269[apigenin, M-H- hexose], 285	C_21_H_19_O_10_-	Apigenin-O-glucopyranoside	Wu et al. (2022)
53	10.28	416.9512	285 [M-H- xylopyranoside]	C_20_H_17_O_10_-	Luteolin 3’-O-xylopyranoside	Wu et al. (2022)
54	10.49	582.9671	431[M-H- galloyl], 341[M-H- galloyl-90], 311[M-H- galloyl—120], 269.05, 169	C_28_H_23_O_14_-	Vitexin 2’’-O-gallate	Singh et al. (2016)
55	11.38	284.9637	151[Ring A fragment], 133	C_15_H_9_O_6_-	Luteolin	Wu et al. (2022)
56	11.41	593	447, 285,183	C_27_H_29_O_15_^−^	kaempferol-3-O-rutinoside	El Sayed et al. (2020)
57	12.39	269	251,225,197,149	C_15_H_9_O_5_-	Apigenin	Wu et al. (2022)
58	13.57	271	253 (M-H-H2O),251,174	C_15_H_11_O_5_^−^	Naringenin	Wu et al. (2022)
59	18.0738	469.2962	317[ M- H- galloyl], 169 [gallic acid], 125	C_22_H_13_O_12_-	Myricetin-3-O-gallate	Singh et al. (2016)
60	21.40	255.1595	227 [M-H-CO], 213 [M-H-C_2_H_2_O], 211 [M-H-CO_2_], 187 [M-H-C_3_O_2_], 151[Ring A, RDA]	C_15_H_11_O_4_-	Pinocembrin	Liu et al. (2005), Mena et al. (2012)
*Anthocyanins*						
61	9.01	482.9747	285[M-H-hexose]	C_21_H_20_ClO_11_^−^	Chrysanthemin	El-Aguel et al. (2022)
62	9.49	284.9818	267[M-H- H_2_O], 257 [M-H-CO], 161	C_15_H_10_O_6_^−^	Cyanidin	Wu et al. (2022)
63	11.63	576.9651	269	C_30_H_25_O_12_^−^	Procyanidin B2	Wu et al. (2022)
*Fatty acids*						
64	9.68	327.12820	309,229,211,171	C_18_H_31_O_5_-	Trihydroxy octadecdienoic acid	Wu et al. (2022)
65	10.30	329.1612	229, 211, 171	C_18_H_33_O_5_-	9,12,13-Trihydroxy-15- octadecenoic acid	Wu et al. (2022)
66	14.91	271.0329	225	C_16_H_31_O_3_-	16-hydroxyhexadecanoic acid	Wu et al. (2022)
*Miscellaneous*						
67	9.89	434.9729	297, 273 [M-H- hexose], 167	C_21_H_23_O_10_-	Phlorizin Di chalcone derivative	Wu et al. (2022)
68	12.43	487.18368	469 [M-H-H_2_O], 457 [M-CH_2_O], 439 [M-H_2_O-CH_2_O], 421 [M-2H_2_O-CH_2_O]	C_30_H_47_O_5_-	Asiatic acid	Wu et al. (2022), Xia et al. (2015)
69	14.56	275.1101	257[M-H- H_2_O], 247[ M-H-CO], 229[M-H- H_2_O-CO] , 219, 203, 191	C_13_H_7_O_7_-	Pentahydroxydibenzo-pyran-one	Singh et al. (2016)
70	15.01	356.9061	342[M-H-CH_3_], 151[M-H-C_12_H_14_O_3_], 136 [M-H-CH_3_-C_12_H_14_O_3_]	C_20_H_21_O_6_^−^	Pinoresinol Lignans/ Phenylpropanoids	Mena et al. (2012)
71	15.06	291.1137	247[M-H- CO_2_], 203 [M-H- 2CO_2_], 177	C_13_H_7_O_8_-	Brevifolin carboxylic acid Isocoumarin	Fischer et al. (2011)
72	15.41	473.1814	473	C_30_H_49_O_4_-	Punicanolic acid	Wu et al. (2022)
73	22.86	455.1906	455	C_30_H_47_O_3_-	Ursolic acid, Oleanolic acid	Wu et al. (2022)

#### Compounds isolation

**Fig. 1:**
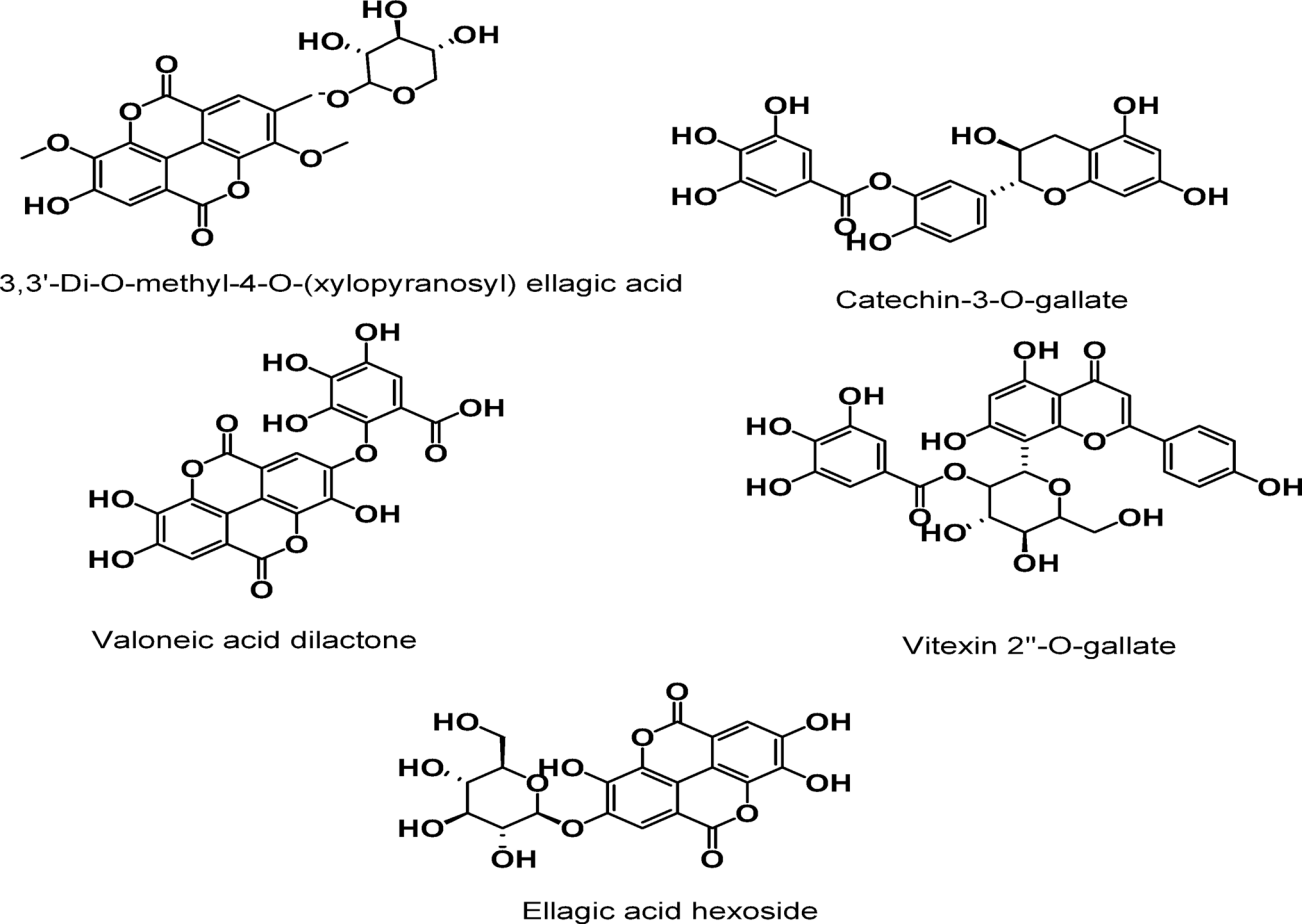
Scheme for isolated compounds from *Punica granatum* L. leaves

The structures of isolated compounds are illustrated in Fig. 1. Table 3 depicted the estimated amount of the isolated compounds (mg/g dry extract) in the PGEL.

##### Compound 1 (peak 43 in LC/MS-MS) 3,3’-Di-O-methyl-4-O-(xylopyranosyl) ellagic acid

It was isolated as white crystalline solid (Rf = 0.51). ^1^H-NMR (400 MHz, DMSO, δ, ppm) recorded two aromatic protons at: 7.50 (H-5, s, 1H, ), 7.71 (H-5’, s, 1H). Di methyl groups’ protons at C-3 and C-4 gave peaks at 4.01 and 4.03, respectively (s, 6H). Xylose protons: 5.11 (H-1″″, d, 1-H), 3.30 (H-2″, s, 2-H), 3.28 (H-3”, s, 1-H), 3.35(H-4″, s, 1H), 3.79 (H-5a″, dd, 1H), 3.29 (H-5b″, dd, 1H). ^13^CNMR (125 MHz, DMSO δ, ppm): 110.78 (C-1), 140.15(C-2), 141.67(C-3), 151.80 (C-4),111.10 (C-5), 113.09 (C-6), 157.24 (C-7), 113.80 (C-1’), 139.90 (C-2’), 140.26 (C-3’), 150.60 (C-4’), 111.40(C-5’), 111.61(C-6’), 157.50(C-7’), 59.67 (3-OCH_3_), 60.14 (3’-OCH_3_), 100.71 (C-1″), 71.87 (C-2”), 75.32 (C-3″), 68.74 (C-4”), 64.86 (C-5”). EI-MS *m/z* gave M^+^ 462 for the molecular formula of C_21_H_18_O_12_. Other major fragments depicted *m/z*: 447 [M-CH_3_], 330 [M-xylose], 315 [M- xylose -CH_3_] and 298. The above spectral data were in accordance with previously stated in literature (Li et al. 1999; Singh et al. 2016). Methylated derivatives of ellagic acid are widely abundant in *P. granatum* plant parts (fruit, flower, seeds and leaves), but this compound is being detected for the first record to our knowledge.

##### Compound 2 (peak 39 in LC/MS-MS) Catechin-3-O-gallate

It was isolated as a white amorphous powder, with Rf value equals 0.81 (chloroform /methanol 90:10 v/v). ^1^H-NMR (400 MHz, DMSO, δ, ppm) showed distinctive protons of 3-O-galloyl moiety at 7.01(H-2”, H-6”, s, 2H), at 6.30 and 6.55 for the aromatic protons (H-6, H-8, d, 2H), 5.16 (H-2, d, 2H), other peaks were: 5.38 (H-3, m,1H), 2.68 (H-4, dd, 2H), ^13^CNMR (125 MHz, DMSO δ, ppm): 78.23 (C-2), 69 .82 (C-3), 23.54(C-4), 151.61(C-5), 103.80(C-6), 158.11(C-7), 101.91(C-8), 155.92 (C-9), 104.81(C-10), 130.71(C-1’), 113.75(C-2’), 145.81 (C-3’), 145.92(C-4’), 116.81(C-5’), 118.13 (C-6’), 120.66 (C-1”), 109.72(C2”, 6”), 145.62(C-3”, 5”), 141.24 (C-4”). EI-MS *m/z* gave M^+^ 442 for the molecular formula of C_22_H_18_O_10_. Other peaks were noticed at *m/z*: 290[M- galloyl], 246[M-galloyl-CO_2_] and 170 (Hamed et al. 2022; Ivanov et al. 2011).

##### Compound 3 (peak 25 in LC/MS-MS) Valoneic acid dilactone

It was obtained as a white amorphous powder, Rf=, ^1^H-NMR (400 MHz, DMSO, δ, ppm) depicted chemical shifts at 6.97 (s, H-5), 7.40 (s, H-5’), δ, 6.99 (s, H-6’’). ^13^CNMR (125 MHz, DMSO δ, ppm): 113.83 (C-1), 104.76 (C-2), 149.72 (C-3), 139.55 (C-4), 108.93 (C-5), 134.68 (C-6), 157.78 (C-7), 111.85 (C-1’), 108.71 (C-2’), 148.73 (C-3’), 139.71 (C-4’), 111.23 (C-5’), 134.83 (C-6’), 158.72 (C-7’), 114.31 (C-l’’), 138.81 (C-2’’), 138.48 (C-3’’), 133.56 (C-4’’), 141.89 (C-5’’), 108.54 (C-6’’), 166.30 (COOH). EI-MS *m/z* gave M^+^ 470 for the molecular formula of C_21_H_10_O_13_ 452(M-H-H_2_O), 442(M-H-CO), 318 (M-H-galloyl). The spectral data was in correspondence with literature (Wyrepkowski et al. 2014) and this compound was previously isolated from *P. granatum* rind (Jain et al. 2012).

##### Compound 4 (peak 54 in LC/MS-MS) Vitexin 2’’-O-gallate

It was isolated as light yellow crystalline powder, Rf = 0.54. ^1^H-NMR (400 MHz, DMSO, δ, ppm) recorded the following peaks: 3.58 (H-6”, m,1H), 3.68 (H-6”, m, H), 3.70–4.79 (m, glucose protons, 4H, ), 4.50(H-1”, d,1H), 6.38 (H-6, s,1H), 6.57(H-3, s, 1H), 6.65 (H-3`, 5`, d, 2H), 7.78 (H-2`, 6`, d, 2H, ), 9.82 (4`-OH, s, 1H), 12.34 (5-OH, s, 1H, ), 7.11 (H-2”’, H-6”’, s, 2H), characterizing protons of galloyl moiety), 4.52 (s, anomeric protons of glucose). ^13^CNMR (125 MHz, DMSO δ, ppm): 163.18 (C-2), 102.92 (C-3), 181.12 (C-4), 160.39 (C-5), 98.29 (C-6), 162.71 (C-7), 105.10 (C-8), 155.56(C-9), 103.68 (C-10), 121.18 (C-1`), 129.23 (C-2`, C-6`), 114.94 (C-3`, C-5`), 161.20 (C-4`), 73.76 (C-1”), 72.50 (C-2”), 78.59 (C-3”), 70.52 (C-4”), 81.80 (C-5”), 62.63 (C-6”), 121.16 (C-1‷), 108.12(C2‷, 6‷), 144.32(C-3‷, 5‷), 140.84 (C-4‷). EI-MS *m/z* gave M^+^ 584 for the molecular formula of C_28_H_24_O_14_ and other fragments were noticed at *m/z*: 432 [M-galloyl], 342 [M-galloyl-90], 312 [M-galloyl-120] and 270. This compound was recognized by comparing the obtained analytical data with that reported in literature (El Sayed et al. 2020; Singh et al. 2016). This compound is detected from *P. granatum* leaves for the first time although it has been identified from other plant species.

##### Compound 5 (Peak 9 in LC/MS-MS) Ellagic acid glucoside

It was isolated as pale yellow amorphous powder, Rf=, ^1^H-NMR (400 MHz, DMSO, δ, ppm) displayed the aromatic protons at 6.91 (3H, 3H’, s, 2H), 4-OH groups appeared at 8.74, 8.89, 9.12 and 12.16 (4H, 5H, 4’H, 5’H, s, 4H), as for glucose protons: 4.67 (H-1”, d, 1H, ), 3.78–4.61 (m, 4H), 3.50 (H-6”, m, 1H), 3.68 (H-6”, m, 1H), 5.01 (OH of glucose). ^13^CNMR (125 MHz, DMSO δ, ppm): exhibited signals at 120.11(C-1,1’), 109.58 (C-5,6,5’,6’), 137.74 (C3,3’), 143.68(C-2,2’,4,4’), 169.83(C-7,7’), 94.42(C-1”), 72.67 (C-2”), 76.82(C-3”), 69.26 (C-4”), 74.73(C-5”), 63.53(C-6”). EI-MS *m/z* gave M^+^ 464 for the molecular formula of C_20_H_16_O_13,_ other fragments at *m/z* 302 [ellagic acid moiety] and 271. The spectral data were compatible with data in literature, and was detected in *P. granatum* leaves and peels extract (El-Aguel et al. 2022; Srivastava et al. 2007).

**Table 3:** Estimated amount of isolated compounds from PGEL

Compound	Quantity (mg/g dry extract)
3,3’-Di-O-methyl-4-O-(xylopyranosyl) ellagic acid	1.31
Catechin-3-O-gallate	1.09
Valoneic acid dilactone	1.11
Vitexin 2’’-O-gallate	0.8
Ellagic acid glucoside	0.71

###  Network pharmacology

**Fig. 2:**
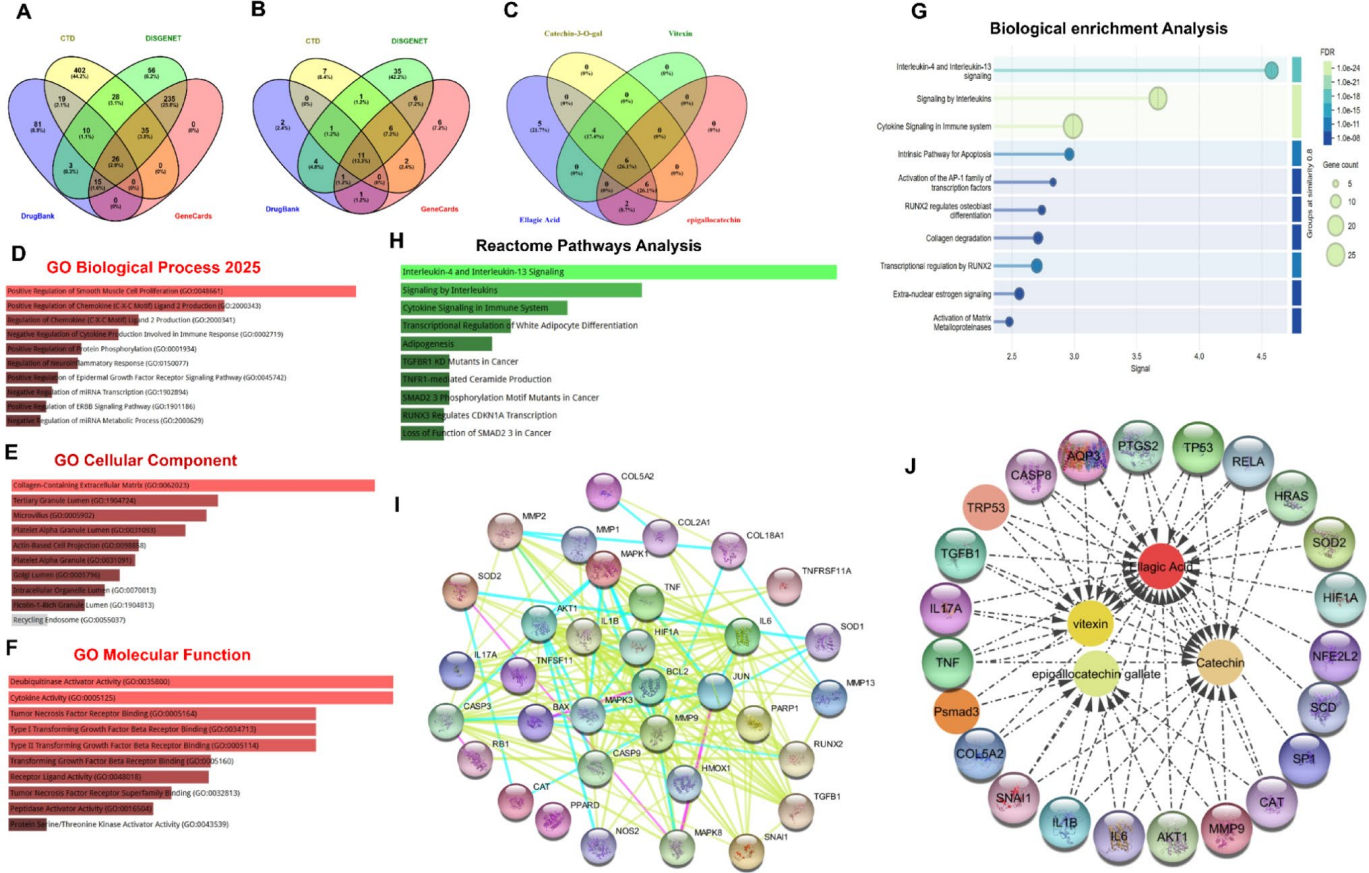
**A**: A Venn diagram illustrating overlapping therapeutic targets derived from CTD, DrugBank, DisGenest, and GeneCards. **B**: A Venn diagram showing overlapping targets from CTD, DrugBank, DisGenest, and GeneCards specifically associated with skin diseases. **C**: A Venn diagram depicting overlapping targets identified from selected compounds. **D**,** E**,** F**: HUB analysis conducted on intersecting targets, with GO enrichment analysis of the top selected targets, covering biological processes, cellular components, and molecular functions. **G**: Biological enrichment analysis performed on HUB genes. **H**: Reactome pathway analysis of HUB genes. **I**,** J**: protein-protein interaction (PPI) network of intersecting targets between compounds and therapeutic targets

By integrating data from GeneCards, CTD, DisGenest, and DrugBank, we identified 910 unique therapeutic targets after removing duplicates (154 from DrugBank, 520 from CTD, 408 from DisGenest, and 311 from GeneCards) (Fig. 2A). Remarkably, 83 targets were common across all databases (20 from DrugBank, 28 from CTD, 30 from DisGenest, and 33 from GeneCards), associated with skin diseases (Fig. 2B), underscoring their potential relevance for treating skin fibrosis. Furthermore, a Venn diagram illustrated the overlapping targets derived from selected compounds (Fig. 2C). For subsequent Gene Ontology (GO) enrichment and KEGG pathway analysis, we prioritized the most central overlapping protein targets.

#### GO enrichment and KEGG pathway analysis

We conducted a GO analysis on 23 key targets, examining cellular components, biological processes, and molecular functions. The top 10 targets with the lowest p-values are shown in Fig. 2D. These proteins are primarily located in cellular compartments, including the Collagen-Containing Extracellular Matrix (GO: 0062023), Tertiary Granule Lumen (GO: 1904724), Microvillus (GO:0005902), Platelet Alpha Granule Lumen (GO:0031093), Actin-Based Cell Projection (GO:0098858), and Platelet Alpha Granule (GO:0031091) (Fig. 2D). The focus on collagen-containing ECM and granule lumens (e.g., platelet alpha granules) reflects the structural and secretory aspects of fibrotic tissue remodeling. For molecular functions, these targets are mainly involved in processes such as Deubiquitinase Activator Activity (GO:0035800), Cytokine Activity (GO:0005125), Tumor Necrosis Factor Receptor Binding (GO:0005164), Type I and Type II Transforming Growth Factor Beta Receptor Binding (GO:0034713, GO:0005114), Transforming Growth Factor Beta Receptor Binding (GO:0005160), Receptor Ligand Activity (GO:0048018), Tumor Necrosis Factor Receptor Superfamily Binding (GO:0032813), and Peptidase Activator Activity (GO:0016504), TGF-β receptor binding and cytokine activity underscore the molecular drivers of fibrosis, while deubiquitinase and peptidase activities hint at regulatory mechanisms (Fig. 2E). The associated biological processes include key functions such as Positive Regulation of Smooth Muscle Cell Proliferation (GO:0048661), Regulation and Positive Regulation of Chemokine (C-X-C Motif) Ligand 2 Production (GO:2000341, GO:2000343), Negative Regulation of Cytokine Production in Immune Response (GO:0002719), Positive Regulation of Protein Phosphorylation (GO:0001934), Regulation of Neuroinflammatory Response (GO:0150077), Positive Regulation of Epidermal Growth Factor Receptor Signaling Pathway (GO:0045742), Positive Regulation of ERBB Signaling Pathway (GO:1901186), Negative Regulation of Fat Cell Differentiation (GO:0045599), and Regulation of Epithelial to Mesenchymal Transition in Endocardial Cushion Formation (GO:1905005) (Fig. 2F).The prominence of TGF-β (ERBB, EGFR) and cytokine/chemokine regulation (IL-4, TNF) aligns with inflammation and fibroblast activation in skin fibrosis. Negative regulation of miRNA suggests enhanced fibrotic gene expression. Using the DAVID database, we identified relevant biological pathways, with the top 20 pathways based on false discovery rate visualized on an online bioinformatics platform (Fig. 2G). Skin fibrosis is characterized by excessive ECM deposition driven by inflammation, cytokine signaling, and TGF-β pathways. The prominence of Interleukin-4 and Interleukin-13 Signaling and Cytokine Signaling in Immune System highlights a strong inflammatory component, as IL-4 and IL-13 drive Th2 responses and fibroblast activation in fibrotic skin diseases like scleroderma. The involvement of TGFBR1 and Smad2-related pathways underscores TGF-β signaling as a key driver of fibrosis, promoting collagen synthesis and myofibroblast differentiation. TNFR1-mediated ceramide production may contribute to initial tissue damage, paving the way for fibrotic repair. Pathways related to adipogenesis, and transcriptional regulation like reflect secondary effects, such as adipose tissue inflammation contribute to skin fibrosis (Fig. 2H)

#### Core targets and PPI network

A protein-protein interaction (PPI) network was developed using identified therapeutic targets, with circular nodes representing individual proteins. The most prominent targets included TNF (Tumor Necrosis Factor), IL6 (Interleukin-6), TGF-β1 (Transforming Growth Factor Beta 1), and COL5A2, indicating greater significance in associated pathways, emphasizing their critical roles in skin fibrosis (Fig. 2I). TNF drives fibroblast activation and collagen synthesis, key processes in fibrosis, and is elevated in fibrotic skin conditions like scleroderma. Compounds such as (+)-Catechin-3-O-gallate and epigallocatechin gallate may reduce TNF-mediated inflammation. TGF-β1, a pivotal fibrosis mediator, promotes fibroblast-to-myofibroblast differentiation and collagen deposition, interacting with compounds like Ellagic acid hexoside and Viteexin, making it a prime target for anti-fibrotic therapies. The PPI network suggests that Ellagic Acid, Viteexin, (+)-Catechin-3-O-gallate, and epigallocatechin gallate may exert anti-fibrotic effects by targeting inflammation (TNF, IL6), extracellular matrix production (TGFB1, COL5A2), oxidative stress (SOD2, CAT), and cell signaling (TP53, AKT1). Ellagic Acid, as the central node, exhibits the broadest influence, positioning it as a promising lead compound for further investigation into skin fibrosis treatments (Fig. 2J)

.

### In vivo findings

#### Effect of PGEL on skin antioxidant and oxidant indicators

**Fig. 3:**
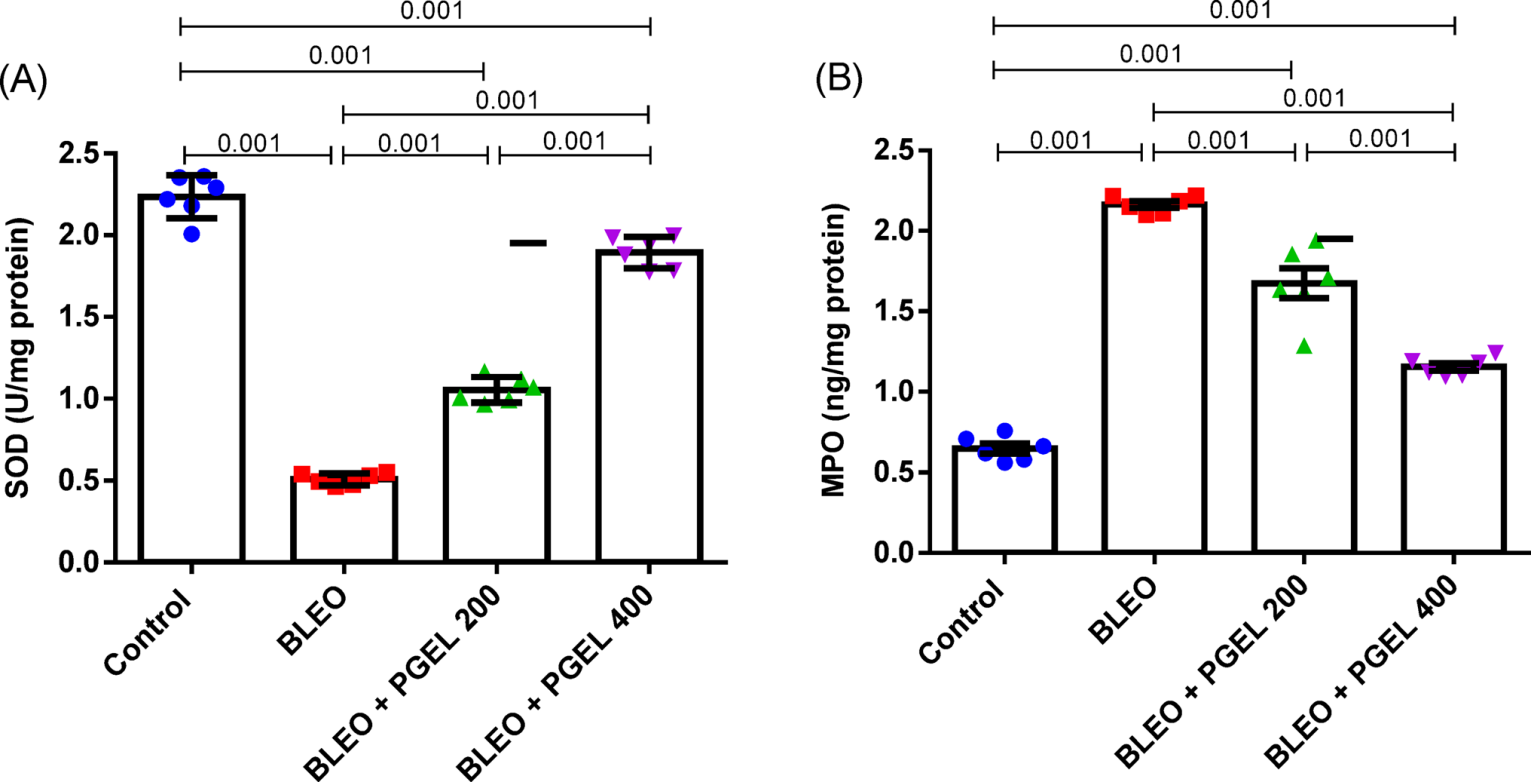
Effect of PGEL on skin antioxidant; SOD (**A**) and oxidant; MPO (**B**) indicators in bleomycin-induced skin fibrosis in rats. Each bar represents mean ± SEM (*n* = 6). SOD; superoxide dismutase, MPO; myeloperoxidase, BLEO; bleomycin, PGEL; *Punica granatum* ethanolic leaf extract

As shown in Fig. 3, BLEO injection for 3 weeks resulted in a significant repression of SOD activity in the dermal tissue when compared to the normal control group. Meanwhile, PGEL administration dose-dependently partially restored the dermal SOD level by 107% and 272% at the dose of 200 and 400 mg/kg respectively, in respective to the BLEO-intoxicated group.

Similarly, a marked elevation of MPO content (2.3 folds) was recorded in the skin tissue of BLEO-challenged group, compared to the un-treated normal rats. Contrary, oral gavage of PGEL at 200 and 400 mg/kg significantly ameliorated the rise of dermal MPO followed bleomycin intoxication by 23% and 47% respectively (Fig. 3).

#### Effect of PGEL on skin inflammatory mediators

**Fig. 4:**
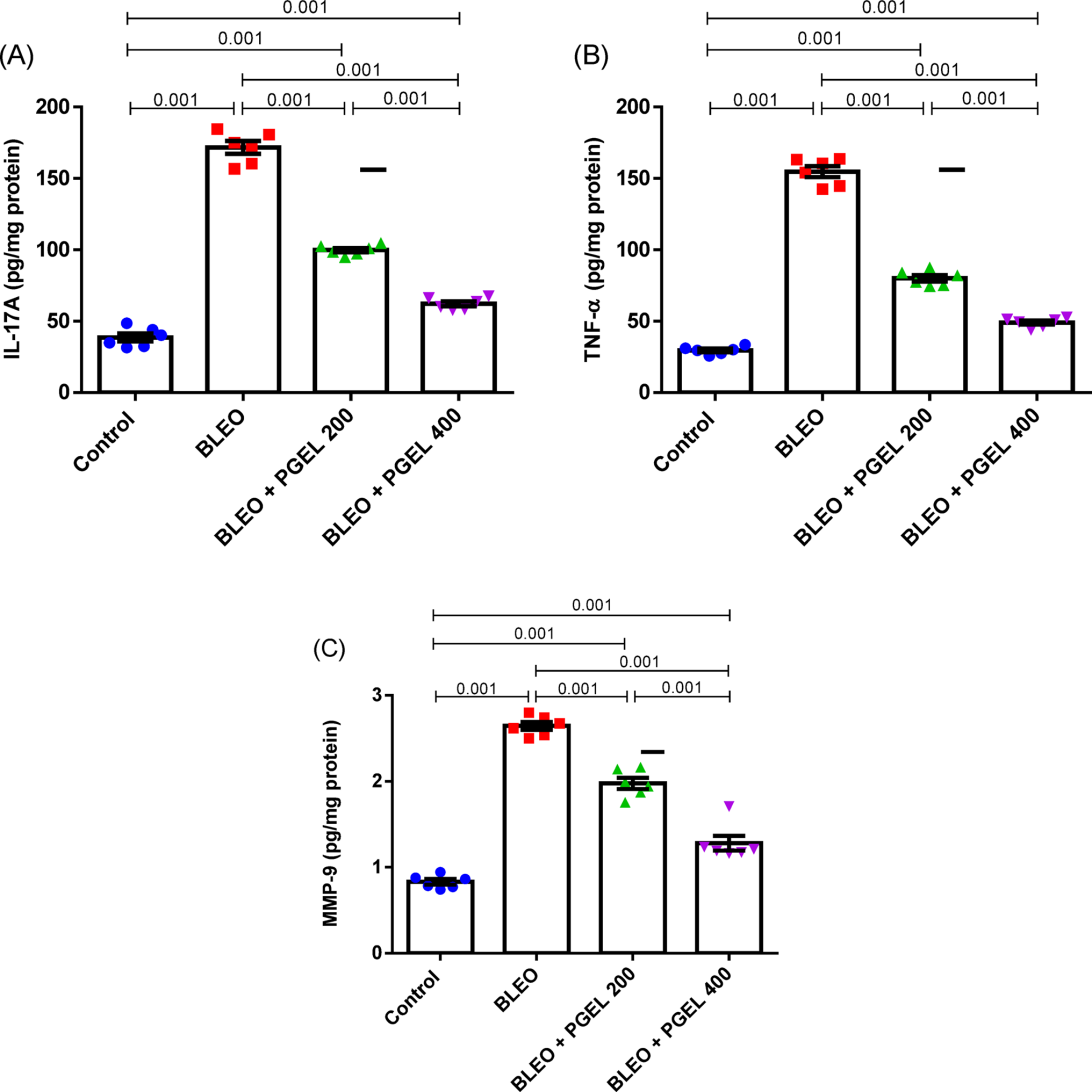
Effect of PGEL on skin inflammatory mediators; IL-17 A (**A**), TNF-α (**B**), and MMP-9 (**C**) in bleomycin-induced skin fibrosis in rats. Each bar represents mean ± SEM (*n* = 6). IL-17; interleukin 17, TNF-α, tumor necrosis factor-α, MMP-9; matrix metalloproteinase 9, and BLEO; bleomycin, PGEL; *Punica granatum* ethanolic leaf extract

A remarkable stimulation of inflammation in the skin tissue of BLEO-injected group was noticed as indicated by significant rise in skin IL-17 A, TNF-α and MMP-9 by 4.4 folds, 5.2 folds and 3.2 folds respectively, in comparison to the control animals (Fig. 4). On the other side, PGEL treatment in a dose-dependent manner (200 and 400 mg/kg) markedly lessened the inflammatory response-triggered by bleomycin intoxication by reducing the skin IL-17 A (42% and 64% respectively), TNF-α (48% and 68% respectively) and MMP-9 (25% and 52% respectively) levels (Fig. 4).

####  Effect of PGEL on skin fibrotic markers

**Fig. 5:**
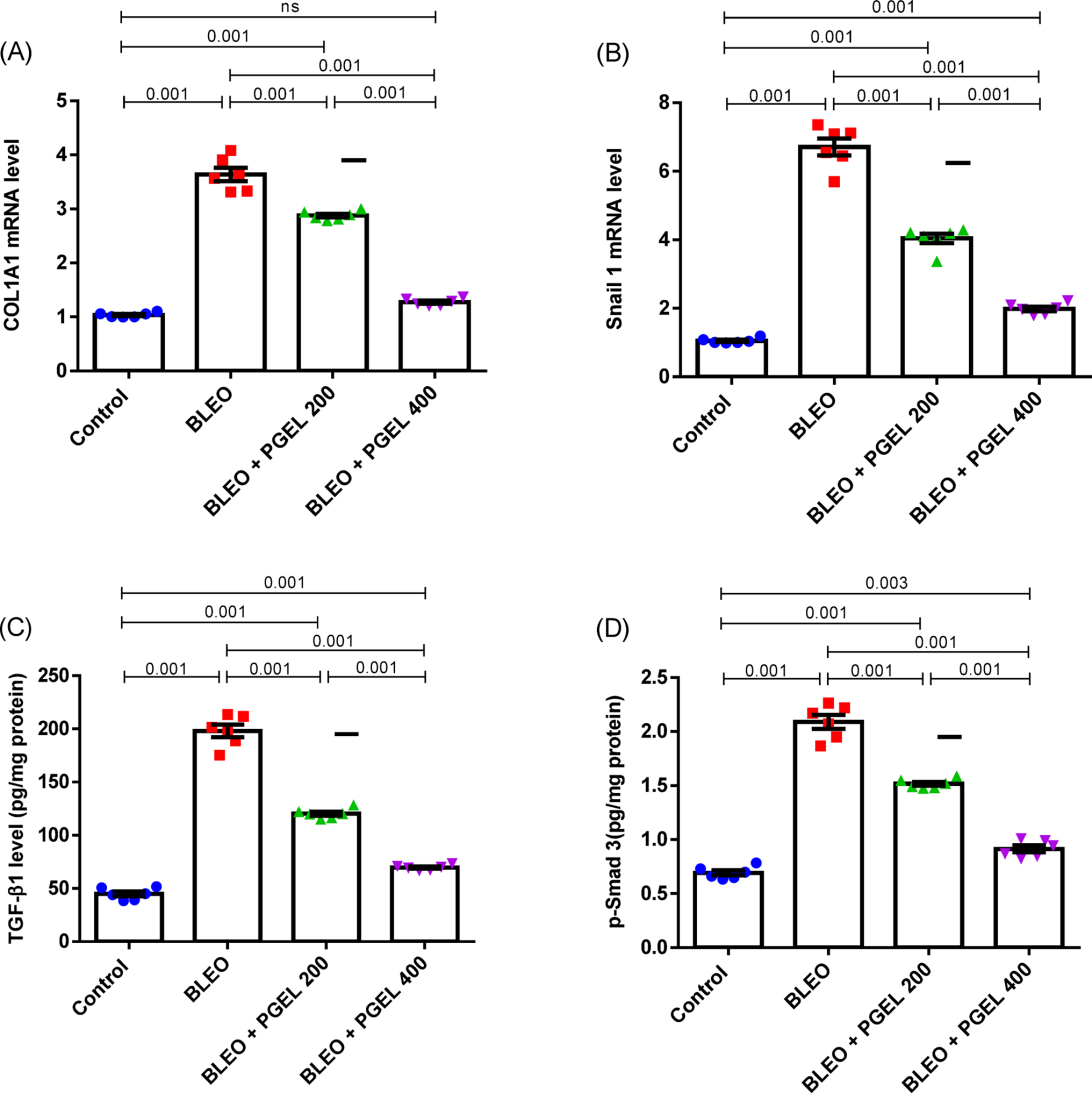
Effect of PGEL on skin fibrotic markers; COLA1 gene (**A**), Snail 1 gene (**B**), TGF-β1 (**C**) and p-Smad 3 (**D**) in bleomycin-induced skin fibrosis in rats. Each bar represents mean ± SEM (*n* = 6). ns; non-significant; COLA1; collagen A1, TGF-β1; transforming growth factor β1, BLEO; bleomycin, PGEL; *Punica granatum* ethanolic leaf extract

Figure 5 demonstrated a significant up-regulation in the COLA1 and Snail 1 gene expression in the skin tissue of BLEO-treated rats, compared to the normal control group. Additionally and in relative to the control animals, bleomycin injection was coupled with a marked boost in the TGF-β1 (4.4 folds) and p-Smad 3 (3 folds) levels in the skin tissue. By contrast, concomitant administration of PGEL at 200 and 400 mg/kg to BLEO-intoxicated rats succeeded to down-regulate the expression of COLA1 and Snail 1 genes, along with diminish TGF-β1 and p-Smad 3 in the skin tissues.

#### Histopathological results

**Fig. 6:**
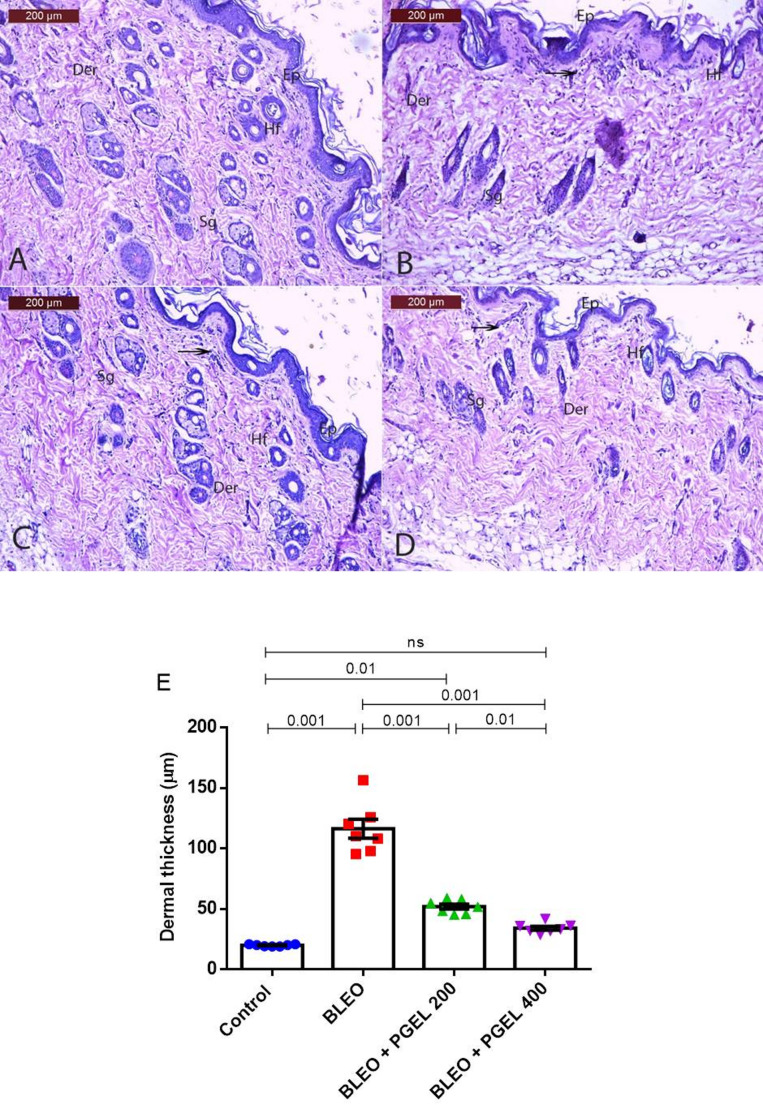
Photomicrographs of sections in skin group stained with H & E showing: **A** Control group showing epidermis (Ep), Dermis (D) many hair follicles (HF) and sebaceous glands (Sg). **B** BLEO group showing definite changes were observed, distorted and discontinuous epidermis with thick epidermis, (Ep), degeneration changes of sebaceous gland (Sg) and hair follicles (HF). Note an increase of inflammatory cells (arrow) in the degenerated dermis (Der), proliferation of fibroblasts (F) and collagen fibres in the dermis and around hair follicles, (arrowhead). **C** BLEO + PGEL 200 group showing more or less normal structure of epidermis (Ep), dermis (Der), reduced thickness of the epidermis, moderate increase sebaceous glands (Sg) and hair follicles (HF). Note slight of inflammatory cells (arrow) in the dermis (Der), moderate proliferation of fibroblasts (F) and collagen fibres (arrowhead). **D** BLEO + PGEL 400 group showing nearly normal structure epidermis (Ep), dermis (Der), sebaceous glands (Sg) and hair follicles (HF), with minimal inflammatory cellular infiltration (arrow), mild proliferation of fibroblasts (F) and collagen fibres (arrowhead). **E** represents the statistical difference of dermal thickness in the studied groups, results are expressed as mean ± SEM (*n* = 7 fields). BLEO; bleomycin, PGEL; *Punica granatum* ethanolic leaf extract

Sections of skin of control group showed that the normal histological structure for skin was formed of epidermis, dermis, many hair follicles and associated sebaceous glands were evident in dermis (Fig. 6A). Histopathological examination of skin of BLEO group showed definite changes were observed, distorted and discontinuous epidermis with thick epidermis, degeneration changes of sebaceous gland and hair follicles. Note an increase of inflammatory cells in the degenerated dermis, proliferation of fibroblasts and collagen fibres in the dermis and around hair follicles with congestion dilated blood vessels (Fig. 6B).

 The BLEO + PGEL200 group showed more or less normal structure of epidermis, dermis, and reduced thickness of the epidermis, moderate increase sebaceous glands and hair follicles. Note slight of inflammatory cells in the dermis, moderate proliferation of fibroblasts and collagen fibres (Fig. 6C).

 The BLEO + PGEL 400 group showed nearly normal structure epidermis, dermis, sebaceous glands and hair follicles, with minimal inflammatory cellular infiltration, mild proliferation of fibroblasts and collagen fibres (Fig. 6D).

Furthermore, significant increase in dermal thickness of BLEO group was documented, compared to the normal control ones. Meanwhile, PGEL administration mitigated the development of dermal thickness in a dose-dependent manner as illustrated in Fig. 6E.

##### Masson’s trichrome-stained results

**Fig. 7:**
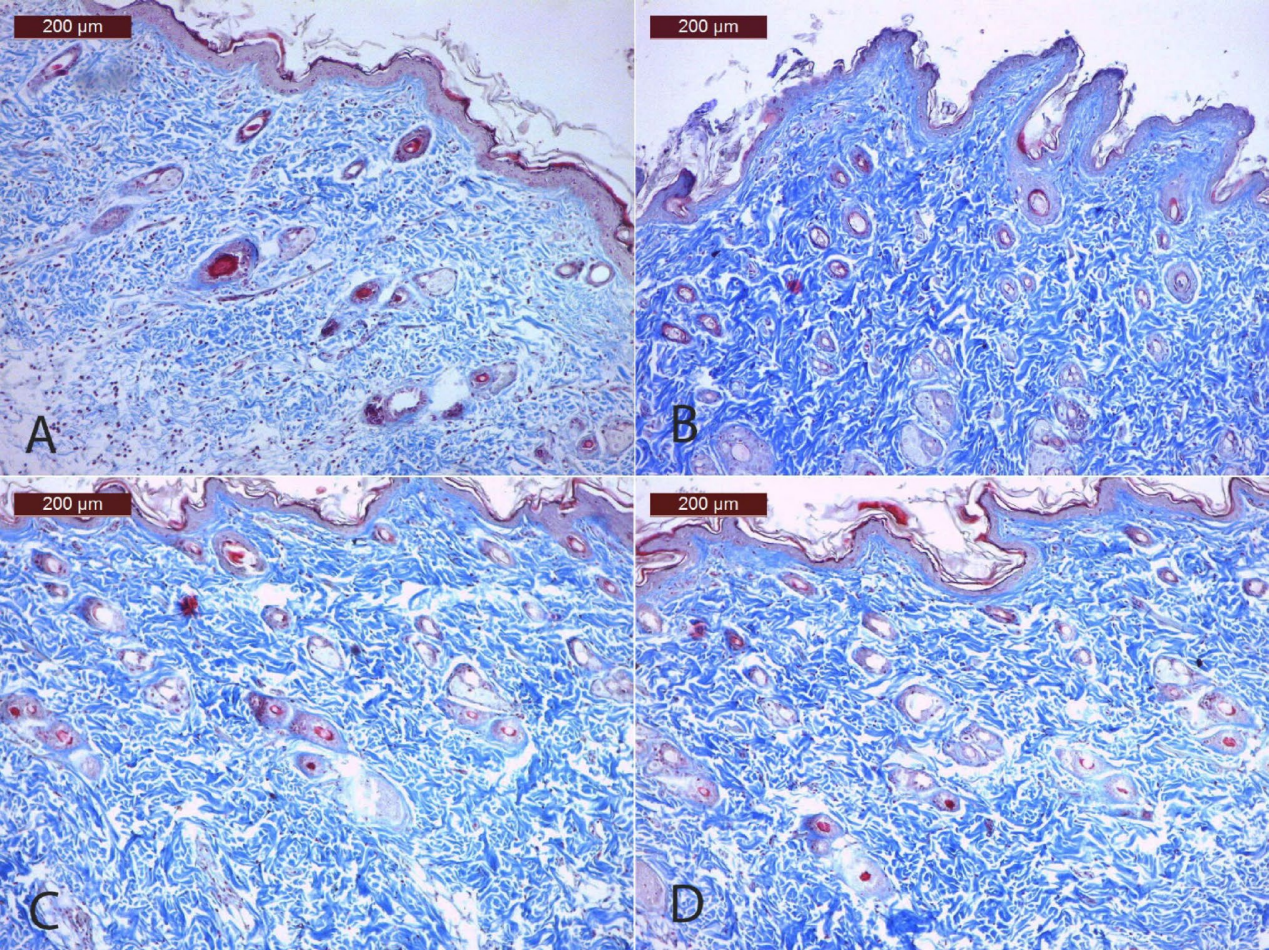
Photomicrographs of sections in skin group stained with Masson’s trichrome stain showing **A**: Control group showing normally appeared loosely arranged collagen fibres in epidermis (E), dermis (D), subcutaneous tissue and around hair follicle. **B** BLEO group showing in the dermis and around hair follicle appeared great amount of collagen fibrils are packed more densely, thicker, irregular and disorganized when compared with those of the control group. **C** BLEO + PGEL200 group showing moderate normal arranged dense collagen fibres of the dermis and around hair follicle. **D** BLEO + PGEL400 group showing normally appeared collagen fibres in the dermis and around hair follicle which appear nearly similar to those of the control group. (Masson’s trichrome x 200). BLEO; bleomycin, PGEL; *Punica granatum* ethanolic leaf extract

Sections of skin of control group showed normally appeared loosely arranged collagen fibres in epidermis, dermis, and subcutaneous tissue and around hair follicle (Fig. 7A). Histopathological examination of skin of BLEO group showed in the dermis and around hair follicle appeared great amount of collagen fibrils are packed more densely, thicker, irregular and disorganized when compared with those of the control group (Fig. 7B). The BLEO + PGEL 200 group showed moderate normal arranged dense collagen fibres of the dermis and around hair follicle (Fig. 7C). The BLEO + PGEL 400 group displayed more apparent collagen fibres arrangement in the dermis and around hair follicle which appear nearly similar to those of the control group (Fig. 7D).

## Discussion

The metabolomics analysis of PGEL revealed the identification of seventy-three compounds belonging to different chemical classes an organic acid, 12 phenolic acids, 4 polysaccharides, 3 amino acids, 19 gallic acid derivatives, 7 ellagic acid derivatives, 14 flavonoids, 3 anthocyanin, 3 fatty acids and 7 miscellaneous groups. In addition, five compounds (catechin-3-O-gallate, 3,3’-di-O-methyl-4-O-(xylopyranosyl) ellagic acid, ellagic acid glucoside, valoneic acid dilactone, and vitexin 2-O-gallate) were isolated and structurally elucidated from PGEL.

Given the significance medicinal impacts of *P. granatum* in improving skin health, this research has been conducted to validate the potential antifibrotic effect of PGEL against skin sclerosis. The key target pathways related to skin disease in particular skin sclerosis were explored through applying network pharmacology. Where, the data obtained from network pharmacology showed that Snail/TGF-β/ COL1A1/Smad signaling pathway, ECM remodeling proteins such as MMP-9, and inflammatory cytokines; TNF-α, and IL-17 A are the key targets in skin sclerosis.

Moreover, further in vivo studies to validate the prophylactic/therapeutic potential of PGEL in the bleomycin-induced experimental scleroderma rat model were established on the level of biochemical, molecular and histopathological investigations. Our results illustrated that subcutaneous injections of bleomycin for 3 weeks (3 times/week) resulted in dermal fibrosis which significantly enhanced by PGEL oral administration. Bleomycin applications induced skin inflammatory cell infiltration, oxidative stress, hyper-activation of myofibroblastic cell that developed dermal thickness which all improved by PGEL treatment at doses 200 and 400 mg/kg in this scleroderma rat model.

As formerly reported, oxidative stress has a central part in the primary molecular mechanism of the SSc (Asano 2018). Hence, we postulated that PGEL could show an anti-fibrotic action through its antioxidant capacity that is highly evidenced in the literature (Viswanatha et al. 2019). In this manner, the SOD and MPO level in the skin tissue were analyzed to assess the oxidative stress between the experimental groups. It was noticed that there was an elevation in the MPO level with noticeable drop of SOD activity of the skin tissue of bleomycin-intoxicated group in relative to the normal control rat. At this theme, it might be believed that a remarkable imbalance ascended from oxidative stress and the relatively deficient antioxidant status upon bleomycin application. Moreover, these findings give a clear evidence of the potent relationship between the overwhelmingly oxidative stress and dermal fibrosis induced by bleomycin, which are in line with previous reports (Roh et al. 2021; Utsunomiya et al. 2022). Bleomycin is recognized for producing ROS, including superoxide and hydroxyl radicals. Multiple research works have demonstrated how oxygen radicals are generated, indicating that the Fenton reaction, with iron serving as a redox agent, may be involved (Bresgen and Eckl 2015). Bleomycin forms complexes with free intracellular iron and, when attached to DNA, directs Fenton-driven OH formation at the DNA. Research has demonstrated that iron is an essential cofactor for prolyl hydroxylase activity (a vital enzyme in collagen synthesis), and that free radicals play a role in prolyl hydroxylase-catalyzed hydroxylated proline formation (Bresgen and Eckl 2015). Thus, lowering the generation of free radicals may help reduce collagen levels by preventing proline hydroxylation. It is believed that heightened production of free radicals plays a significant role in the development of SSc by initiating damage to tissue (Doridot et al. 2019). In patients with SSc, peripheral neutrophils generate elevated ROS levels that correlate with the severity and extent of skin involvement (Doridot et al. 2019). ROS can induce various abnormalities, including damage to endothelial cells and increased platelet activation. This can result in the upregulation of adhesion molecule expression and the secretion of inflammatory or fibrogenic cytokines, such as PDGF and TGF-β. Hence, counteracting ROS production, neutralizing free radicles, or enhancing/preserving antioxidant status are essential ways to mitigate SSc development. A previous study informed the significant role of SOD to reduce dermal sclerosis induced by bleomycin. However, PGEL significantly enhanced SOD level and declined MPO formation that could be ascribed to the antioxidant activities of its constituents.

Matrix metalloproteinases (MMPs) such as MMP-9 are frequently regarded as indicators of skin fibrosis. During the initial phases of the pathological process, a rise in their enzymatic activity leads to considerable alterations in the composition of the ECM. Fibrosis is a multifaceted process marked by the irregular accumulation of ECM, which can result in changes to tissue structure that affects organ function and survival (Asano 2018). Equipped with the complete apparatus for ECM deposition, fibroblasts continuously renew ECM under homeostatic conditions. Crucially, fibroblasts are affected by a range of other cell types, particularly those residing in the tissue undergoing fibrotic changes or professional inflammatory cells recruited to the area (Asano 2018). Soluble mediators of inflammation, particularly cytokines such as TNF-α and IL-17 A, as well as growth factors, play a crucial role in controlling fibroblast migration, proliferation, metabolism, and ECM deposition (Lei et al. 2016). Additionally, T cells are known to have a significant role in the development of SSc. Th17 cells have been demonstrated to be involved in the development of various autoimmune diseases, such as systemic lupus erythematosus, psoriasis, ankylosing spondylitis, and multiple sclerosis (Lei et al. 2016). Former research has also informed the key and central role of Th17 cells in the pathogenesis of SSc through stimulating several inflammatory mediators (Bălănescu et al. 2017). Of these modulators, IL-17 A is the most significant cytokine. Besides Th17 cells, IL17 is produced by various other cell types, including polymorphonuclear cells, macrophages, type 3 innate lymphoid cells, and natural killer cells (Bălănescu et al. 2017). According to Truchetet et al. (Truchetet et al. 2013) and Almanzar et al. (Almanzar et al. 2016), the expression of IL-17 is elevated in the skin and bronchoalveolar fluid of patients with SSc. It was reported that Th17 cells correlate with disease activity in SSc patients, and IL-17 levels enhance fibroblastic activity, leading to greater collagen production (Yang et al. 2008).

Consistent with other studies (Roh et al. 2021; Utsunomiya et al. 2022), our research data clarified that a significant increase in skin TNF-α, IL-17 A and MMP-9 in the bleomycin group, compared to the healthy rats. However, PGEL significantly antagonized the rise of inflammatory cytokines including TNF-α, IL-17 A and MMP-9 in the skin tissues of the PGEL-treated groups, in a dose dependent manner. These findings highly supported the observed decline in MPO activity which has been recognized as inflammatory marker. All these results reinforced the potent anti-inflammatory property of PGEL that could be referred to its phytochemical constituents.

Furthermore, TGF-β is especially viewed as a primary mediator of fibrosis, playing a significant role in the recruitment and trans-differentiation of cell precursors into myofibroblasts. TGF-β is recognized for its crucial involvement in the fibrotic process in SSc, as it stimulates myofibroblasts, fosters perivascular inflammation, and activates immune cells (Lafyatis 2014). Myofibroblasts possess contractile properties linked to the downstream of Smad3, and Snail 1, as well demonstrate a remarkable ability to synthesize and secrete ECM components, including type I and type III collagen, fibronectin, and tenascin, among others (Chen et al. 2016). Once it binds to its receptors, TGF-β fulfills its biological function via the phosphorylation of Smad 2/3 (canonical pathway) (Asano 2020). Moreover, bleomycin induces endothelial cells to produce TGF-β at both the protein and messenger levels, and it promotes collagen synthesis in fibroblasts (Phan et al. 1991). The TGF-β/Smad 3 pathway is thought to play a role in the onset of skin fibrosis caused by bleomycin (Ruzehaji et al. 2016; Utsunomiya et al. 2022). Our research data documented the significant increase in TGF-β and p-Smad 3 levels with upregulation in Snail 1 and COL1AI gene expression in the skin tissue of bleomycin-challenged animals. These results are in line with previous reports (Roh et al. 2021; Utsunomiya et al. 2022). Thus, TGF-β is viewed as a key therapeutic target for SSc. Given that PGEL inhibited TGF-β/p-Smad 3 signaling and the TGF-β-mediated expression of COL1A1and Snail 1 in our study, we hypothesized that it could have therapeutic potential for SSc treatment.

Simultaneously, the histopathological examination of the skin tissue of bleomycin group illustrated profound distorted and discontinuous epidermis with thick epidermis, degeneration changes of sebaceous gland and hair follicles, with marked increase of inflammatory cells in the degenerated dermis, proliferation of fibroblasts and collagen fibres in the dermis and around hair follicles with congestion dilated blood vessels. Conversely, PGEL administration attenuated all these noticed alterations with considerable normalization of dermal thickness at the high dose level (400 mg/kg) which validated by the morphometric analysis, reinforcing PGEL’s anti-fibrotic potential.

Collectively, the observed antioxidant, anti-inflammatory and anti-fibrotic action of PGEL could be highly ascribed to the biological activities of its diverse phytochemical constituents mainly belonging to flavonoids, proanthocyanidins and phenolics classes as reported in our study. For example, gallic acid and its derivatives such as valoneic acid dilactone, catechin-3-O-gallate, and vitexin 2’’-O-gallate possess multiple properties such as anti-inflammatory, and antioxidant (Cláudio et al. 2012). Moreover, ellagic acid and their derivatives (hydrolyzable tannins; ellagitannins) e.g.: ellagic acid glucoside, 3,3’-Di-O-methyl-4-O-(xylopyranosyl) ellagic acid, isolated from the PGEL in the study, attributed as cellular anti-oxidant by motivating the antioxidant enzymes expression, thereby alleviating the oxidative stress (Hanga-Farcas et al. 2025). On the other hand, ellagitannins showed anti-inflammatory activities in humans and animals (Usta et al. 2013). However, catechin provides scavenging radical and anti-oxidative activities (Ranjha et al. 2023).

## Conclusion

In summary, this study employs a multifaceted approach that includes LC/MS, network pharmacology, and experimental validation to investigate the underlying processes and pharmacological effects of the primary PGEL’s components in skin sclerosis. Notably, PGEL administration (200 and 400 mg/kg) efficiently lowers oxidative stress and inflammation, fortifying its anti-fibrotic action, and consequently alleviates skin fibrosis brought on by bleomycin in rats. The results provide insights for therapeutic application and support the use of PGEL for SSc. However, conducting additional research could be essential to provide deep insights in to other potential mechanisms through which PGEL and its isolated compounds could exert its anti-fibrotic effects. Future quantitative LC–MS/MS analyses could be followed to comprehensively characterize the quantitative composition of the extract.

## Data Availability

Data is provided within the manuscript.

## Abbreviations

COL1A1: Collagen 1 Type A1
ECM: Extracellular matrix remodeling
GAPDH: Glyceraldehyde-3-phosphate dehydrogenase
IL-17 A: Interlekin-17A
LC-MS: Liquid chromatography-mass spectroscopy
MMP-9: Matrix metalloproteinase-9
MPO: Myeloperoxidase
*P. granatum*: *Punica granatum*
PGEL: *P. granatum* ethanolic leaf extract
SOD: Superoxide dismutase
SSc: Systemic sclerosis
TGF-β1: Transforming growth factor-β1
TNF-α: Tumor necrosis factor α
